# Common Peroneal Nerve Palsy at the Level of Proximal Fibula After Total Hip Arthroplasty: A Case Report

**DOI:** 10.7759/cureus.30741

**Published:** 2022-10-27

**Authors:** Asim M Makhdom

**Affiliations:** 1 Orthopaedic Surgery, King Abdulaziz University Faculty of Medicine, Jeddah, SAU

**Keywords:** hip surgery, total hip arthroplasty: tha, common peroneal neuropathy, s: foot drop, hip and knee replacement

## Abstract

Sciatic nerve injury is a well-known devastating complication and is the most commonly involved nerve after total hip arthroplasty (THA). Most of these injuries occur at the level of the hip and surgery site rather than a distal location. In this case report, a 62-year-old male presented with common peroneal nerve (CPN) palsy at the level of the knee immediately after undergoing left THA via posterior hip approach. This likely occurred due to direct compression during the surgical positioning or intraoperative leg manipulation. It was associated with excruciating uncontrolled neuropathic pain around the ipsilateral lateral leg and foot and absence of motor function. The patient's THA was performed elsewhere, seven weeks prior to his presentation to us. His clinical examination and electromyographic (EMG) findings confirmed focal peroneal nerve entrapment around the neck of the fibula. An urgent distal peroneal nerve decompression was performed followed by a dramatic improvement in the pain. Consequently, the patient discontinued all pain medications within three days after the decompression. On follow up, he demonstrated remarkable improvement in his motor and sensory functions. In conclusion, direct or indirect CPN injury at the level of the knee is extremely rare after THA. Early distal peroneal nerve decompression after THA can be beneficial in selected patients based on the clinical presentation and EMG findings.

## Introduction

Primary total hip arthroplasty (THA) is one of the most successful orthopaedic procedures. The number of patients undergoing total hip arthroplasty [1] is expected to grow to 1.6 million by 2030 in the United States. Sciatic nerve injury has been cited [2, 3] as a rare complication (approximately 0.5%) that leads to loss of motor (foot drop) and sensory function of the peroneal division of the sciatic nerve. This complication can be a result of various etiologies [4]: at the level of the hip, such as excessive nerve stretch due to lengthening, retraction injury, cement extravasation, acetabular screws misplacement, postoperative hematoma, and direct thermal injury or laceration. The current management of sciatic nerve injury after total hip arthroplasty is treating the underlying cause (if found), removing compressive dressing, extending the hip and flexing the knee to ease the tension on the nerve, supportive conservative measures including foot ankle orthosis to prevent ankle contractures. If the patient shows no recovery, electromyography (EMG) is typically performed at six weeks or 12 weeks. It has been reported that 30% to 50% of patients may have a spontaneous recovery within one year. In those patients who had no recovery, previous small series [5-7] have shown some benefits of sciatic neurolysis when compared to conservative management. However, in extremely rare cases, the level of injury can occur at the level of the knee and fibular tunnel after THA [8]. In this case report, the possible etiologies of such complications and management approaches were discussed. To the best of our knowledge, this would be the second report in the English medical literature for a common peroneal nerve (CPN) palsy at the level of the knee and would be the first report to show the potential benefits of early (within eight weeks after palsy) distal peroneal nerve decompression (PND)around the fibular tunnel after peroneal nerve palsy secondary to THA. 

## Case presentation

**Figure 1:**
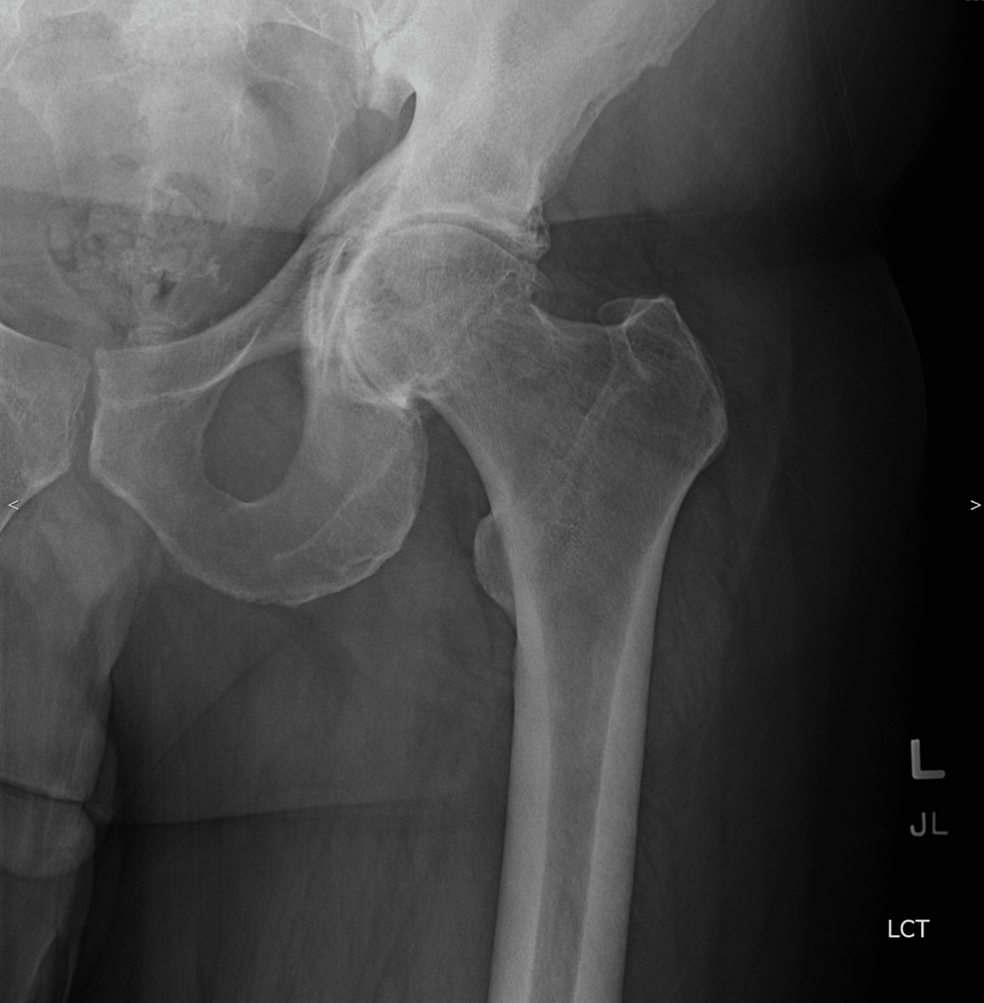
Preoperative anteroposterior radiograph of the left hip showing an advanced left hip primary osteoarthritis.

**Figure 2:**
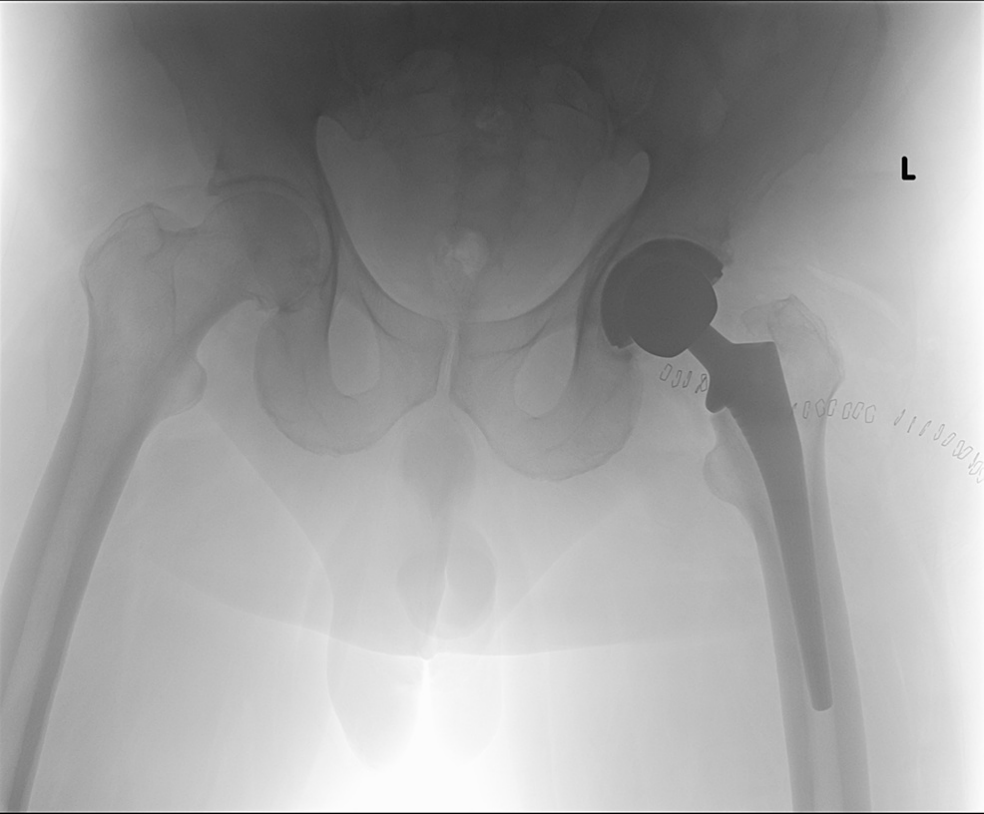
Postoperative immediate pelvis anteroposterior radiograph showing cementless femoral and acetabular components without evidence of overlengthening of the left lower extremity.

A 62-year-old male patient presented to the clinic with a history of left THA, performed elsewhere, for a severe primary hip osteoarthritis. He had the THA performed via posterior hip approach seven weeks prior to his presentation. The surgical procedure was performed without any reported intraoperative complications. However, immediately postoperatively, the patient experienced a loss of motor and sensation function of the common peroneal nerve (CPN) distribution. Dorsiflexion of the ankle (tibialis anterior) and extension of the big toe (extensor hallucis longus) was graded as 0 out of 5 according to the Medical Research Council (MRC) scale. Complete loss of sensation of the lateral side of his leg and dorsal 1st web space was reported. The radiographic hip images did not show any evidence of a possible underlying cause of sciatic nerve injury, such as protruding screws or cement extravasation since the patient had cementless acetabular (without screws) and femoral components (Figures 1, 2)

The patient had not perceived any leg length discrepancy (LLD) before and after his THA procedure. His LLD was measured as 4 millimeters based on his postoperative X-rays. This was calculated and measured as described [9] previously. The left hip ultrasound did not show any evidence of compressing hematoma or collection. His treating surgeon initiated conservative measures including the application of ankle foot orthrosis. Within the first six weeks, the patient progressively developed severe persistent uncontrolled neuropathic pain around the neck of the fibular, lateral leg that radiated down to the dorsal foot. He presented to the office seven weeks after his initial surgery. The clinical examination and electromyography (EMG) results confirmed focal entrapment of the common peroneal nerve with loss of motor and sensory function. 

At the time of the presentation, the patient required high doses of narcotics as well as high doses of gabapentin in order to minimize his pain. The patient was so devastated that he asked for amputation as a possible option to relieve his pain. A thorough review of his medical records (including the operative report) and postoperative radiographic images showed no underlying direct cause of sciatic nerve injury such as laceration or lengthening of the ipsilateral limb. A Tinel's sign [10] at the level of the fibular was positive and the diagnostic lidocaine injection around the fibular neck significantly improved the patient's pain. Based on the clinical examination and EMG results, a decision was made to decompress the peroneal nerve around the neck of the fibula.

**Figure 3:**
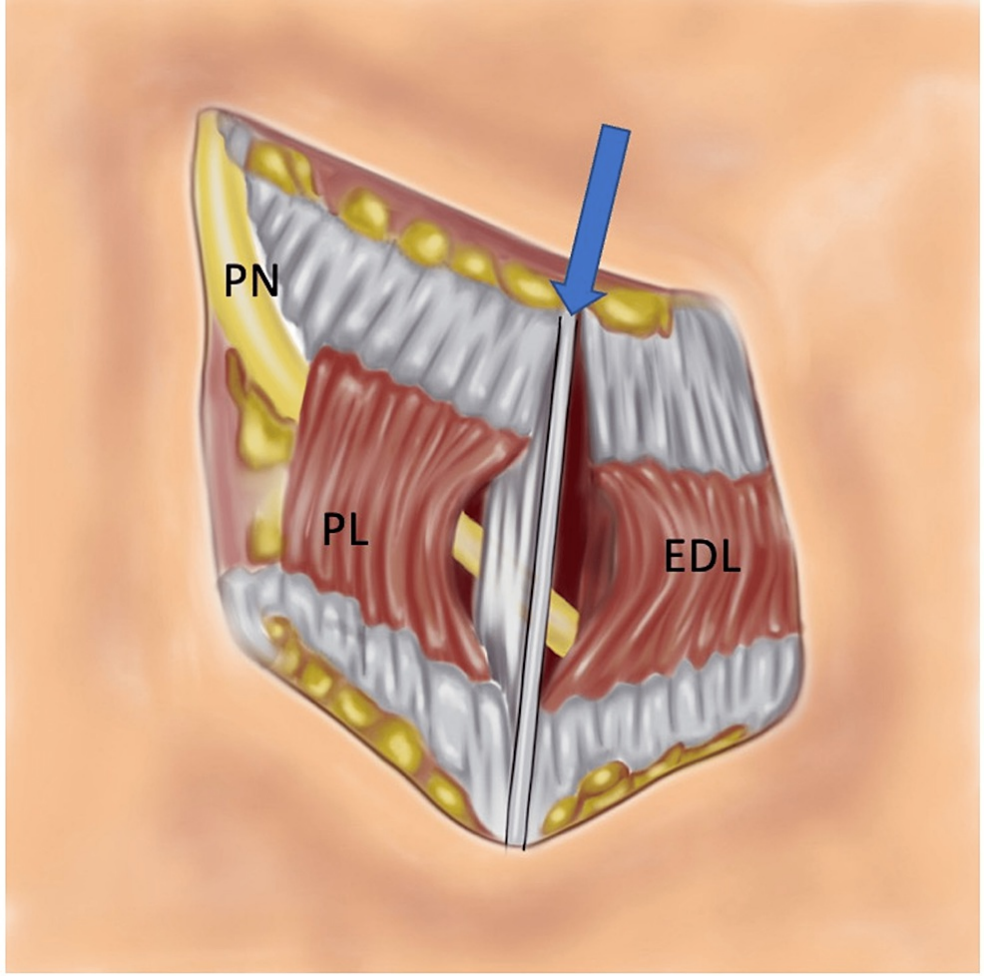
Drawing to show the vertical intermuscular septum (arrow) between the peroneus longus (PL) and the extensor digitorum longus (EDL) muscle that acts as a compression point on the peroneal nerve (PN). Image credit: Asim Makhdom

The peroneal nerve decompression was performed through an oblique 6-cm incision just distal to the fibular neck to follow the course of the peroneal nerve. The knee was positioned at 30 degrees of flexion to ease the tension on the nerve. Once the tourniquet was inflated, the skin and subcutaneous tissues were incised. Meticulous dissection was performed to reach the fascial layer that is covering the peroneal nerve (PN). Careful dissection was performed to avoid damaging the lateral sural nerve which was found at the posterior aspect of the incision. Prior to incising the fascia, the nerve was easily palpable just below the neck of the fibular head. In this case, the overlying muscular fascia around the PN was thick, fibrotic, and tight and acting as a compression point. No evidence of nerve bruising was noted intraoperatively. This fascia was incised and intermuscular septae that typically act as compression points (Figure 3) were released.

The superficial and deep fascia of the peroneus longus muscle and the vertical intermuscular septum between the peroneus longus and the extensor digitorum longus muscles were incised and decompressed. Once these septae were released, the tourniquet was deflated and excellent hemostasis was obtained by the means of bipolar electrocautery. This was followed by subcutaneous and skin closure. The tourniquet time was 26 minutes. Intraoperative neuromonitoring was not used in this case but its use can be helpful in providing better intraoperative feedback for future cases. Immediate postoperative findings showed complete improvement of his neuropathic pain with improved sensation along the peroneal nerve distribution. The extensor hallucis longus (EHL)motor function was graded as 3 out of 5 while ankle dorsiflexion was graded as 0. Follow-up intervals at one week, three months, six months, and one year showed progressive improvement in his motor function with the latest EMG showing tibialis anterior muscle power (dorsiflexion of the ankle) graded as 3 out of 5 at one year of follow up. The patient has discontinued all narcotics and gabapentin after three days following the peroneal nerve decompression. 

## Discussion

Sciatic nerve palsy at the level of the hip and CPN palsy at the level of the knee have similar postoperative presentations after THA. The current management of sciatic nerve injury after THA is mostly conservative with supportive measures. In a systematic review, the spontaneous recovery rate was found to be 33% [7]. Some individual studies have reported it as 50% [3] within a year after surgery. The peroneal division is more sensitive and vulnerable in these circumstances, can lead to dysfunctional gait, and is highly associated with poor outcomes. In this case report, the patient had CNP compression at the level of the knee, and early PND led to significant improvement in both motor and sensory function of the nerve and complete recovery of the neuropathic pain. 

The concept of performing distal PND after CPN injuries secondary to THA is not new. Wilson et al [8] have examined 37 patients who underwent THA and sustained sciatic nerve injury. Patients underwent PND after a median one-year follow-up after sciatic nerve injury and THA. Sixty-five percent of patients have recovered ankle dorsiflexion with MRC>3. The authors found the presence of Tinel's sign as a predictor of success. In this report, the patient had a positive Tinel's sign as well as a positive response to the lidocaine diagnostic injection at the level of the fibular neck. Although the patient did not have a full ankle dorsiflexion recovery, his immediate postoperative pain relief with motor and sensory improvement emphasized the potential benefits of performing early decompression in these eligible patients. 

There are several hypotheses that can explain why the distal peroneal nerve site is involved after THA. It’s possible that some patients have a preexisting subclinical focal entrapment of the PN around the head of the fibula. Having a proximal lesion due to retraction injury or minor nerve stretch during hip manipulation can result in a clinical deficiency. This supports the concept of the “double crush phenomena” where two superimposed nerve insults can result in a peroneal nerve injury. Another theory is that some patients have poor glide of PN around the neck of the fibular due to a lack of range of motion preoperatively secondary to hip/knee osteoarthritis. The leg manipulation during the surgery or the patient’s positioning under anesthesia may result in a clinical deficit. Park et al [3] have reported one patient out of 31 who developed common peroneal nerve palsy due to a direct compression at the level of the knee during THA. Finally, in some patients with preexisting lumbar spine pathology and peroneal nerve compression due to a limited preoperative lack of motion or immediate postoperative swelling, a clinical deficit might also be encountered. Thus, magnetic resonance imaging of the lumbar spine can be helpful to detect a compression at a higher level. 

The treatment algorithm for a sciatic nerve injury after THA has been extensively reported but it varies between institutions. The first immediate step is to initiate conservative measures such as providing ankle foot orthrosis. This is initiated while looking for an underlying cause. Acetabular screws misplacement, cement extravasation, or hematoma collection compressing the nerve are possible etiologies. Immediate radiographic images, ultrasound or magnetic resonance images can help to rule out these etiologies. Additionally, over-lengthening (more than 2 cm) [11]) of the lower extremity can result in sciatic nerve damage (commonly its peroneal division), and therefore, pre- and postoperative radiographs and clinical examination are necessary to estimate the LLD. If no underlying cause is found and/or no direct laceration of the nerve is expected, conservative measures are continued, and baseline EMG is requested after six weeks.

**Figure 4:**
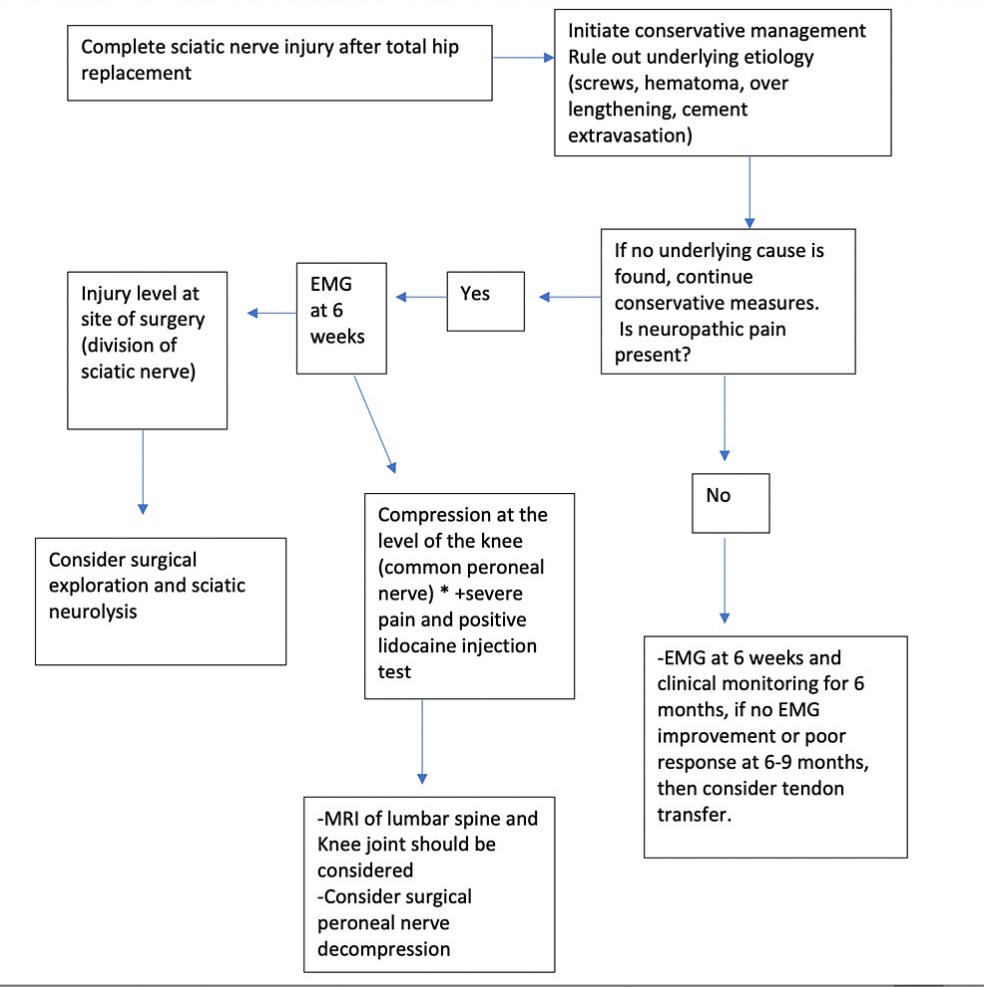
Our current treatment approach of complete sciatic nerve palsy after total hip arthroplasty with and without neuropathic pain. EMG: electromyography

Some authors [5] suggested that patients with severe neuropathic pain will benefit from sciatic nerve exploration and neurolysis if the EMG suggested an injury at that level. In this report, the patient has significant neuropathic pain and the EMG showed that the level of compression was around the neck of the fibula which is extremely rare after a THA. A positive lidocaine test and positive Tinel's sign were positive predictors to achieve a successful outcome. The summary of our current treatment approach is illustrated in Figure 4. 

## Conclusions

In conclusion, this case illustrates the potential benefits of early distal peroneal nerve decompression in such rare clinical scenarios when the level of the injury is at the proximal fibula. The decision should be supported by clinical examination and EMG findings.
